# The Impact of Body Surface Area on Morpho-Functional and Cardiometabolic Parameters in a Large Cohort of Olympic Athletes: Distinct Bodies, Distinct Physiology

**DOI:** 10.3390/jfmk10040405

**Published:** 2025-10-18

**Authors:** Giuseppe Di Gioia, Maria Rosaria Squeo, Armando Ferrera, Lucrezia Macori, Margherita Rigillo, Raffaella Spada, Antonio Pelliccia

**Affiliations:** 1 Institute of Sports Medicine and Science, National Italian Olympic Committee, Largo Piero Gabrielli, 1, 00197 Rome, Italy; dottgiuseppedigioia@gmail.com (G.D.G.); mariarosaria.squeo@coni.it (M.R.S.); armando.ferrera95@gmail.com (A.F.); lucreziamacori@gmail.com (L.M.); ext_raffaella.spada@coni.it (R.S.); 2 Department of Movement, Human and Health Sciences, University of Rome “Foro Italico”, Piazza Lauro De Bosis, 15, 00135 Rome, Italy; 3 BIOMETRA Department, University of Milan, 20129 Milan, Italy; dottoressa.margherita.rigillo@gmail.com

**Keywords:** body surface area, Olympic athletes, cardiac remodeling, cardiometabolic risk, sports classification

## Abstract

**Background:** Body surface area is a key determinant of cardiac morphology and function, but it is often underestimated in the interpretation of athlete’s cardiac phenotypes. **Aims:** This study aimed to assess the role of anthropometric characteristics and whether particularly high vs. low body surface area (BSA) is associated with distinct morpho-functional and cardiometabolic features in elite athletes. **Methods:** We retrospectively included 2518 Olympic athletes. All underwent a pre-participation screening, including physical examination, ECG, blood analysis, echocardiography, and cardiopulmonary exercise testing. Participants were grouped by sex-specific BSA percentiles: Group A (<5th percentile), Group B (25th–75th), and Group C (>95th percentile). Functional, echocardiographic, and cardio-metabolic parameters were compared among groups. **Results:** In male athletes, Group C showed higher resting systolic blood pressure (123.8 ± 10.4 mmHg) than Group B (117.4 ± 9.6, *p* < 0.0001) and Group A (110.4 ± 13, *p* < 0.0001), and a higher prevalence of dyslipidemia (31.7% vs. 11.1% in Group B and 4% in Group A, *p* = 0.031). Despite greater LVEDD (59 ± 3 mm in Group C vs. 55 ± 2.9 in B and 51.1 ± 3.1 in A, *p* < 0.0001) and LV mass (*p* < 0.0001), functional performance was lower in Group C, with VO_2_ max/kg of 35.2 ± 13.2 mL/min/kg vs. 44 ± 7.1 in B, and 47.8 ± 7.3 in A (*p* < 0.0001). Similar trends were observed in females for morpho-functional parameters, though lipid profiles did not significantly differ among groups (*p* > 0.05). **Conclusions:** Anthropometric traits significantly influence the cardiovascular and metabolic phenotype of elite athletes. Our findings support the integration of anthropometric profiling into the routine cardiovascular assessment of athletes, especially those at the extremes of body size, to better interpret physiological adaptations and risk profiles.

## 1. Introduction

Olympic athletes represent a highly select population, characterized by intense and prolonged training regimens, exceptional physical performance, and distinct physiological adaptations [1,2]. Nevertheless, significant heart variability exists; despite a similar status of top-level competitors and high achievements, athletes differ in terms of cardiac remodeling, functional capacity, and metabolic profile [3,4,5,6,7,8]. In 2020, the European Society of Cardiology (ESC) proposed a widely used classification that groups athletes into four categories: skill, power, mixed, and endurance [9].

This classification has historically aimed to cluster athletes with similar profiles in terms of cardiac remodeling patterns, functional parameters, sport typology, training load, and even cardio-metabolic characteristics [9,10,11]. While pragmatic and clinically helpful, this approach assumes that the type of sport represents a major determinant for cardiac remodeling, implying a measure of homogeneity within each sport category [9].

However, anthropometric parameters, such as body surface area (BSA), substantially contribute to the variability in cardiac size and function, as previously reported [12].

Anthropometric traits may significantly affect the extent of cardiovascular structure, functional performance, and cardiometabolic risk.

With this perspective, we sought to analyze cardiac remodeling in elite athletes from a new perspective, shifting the focus from sport discipline to body size, in order to search for new insights into the physiological and metabolic adaptations of elite athletes across the body-size spectrum.

Therefore, the aim of this study was to provide a comprehensive description of the morpho-functional and cardiometabolic profiles of a large cohort of Olympic athletes, divided according to their BSA, to explore, in particular, whether extreme anthropometric characteristics are associated with distinct cardiac features.

## 2. Materials and Methods

### 2.1. Setting

The Institute of Sport Medicine and Science in Rome, division of the Italian National Olympic Committee, is responsible for conducting medical evaluations of athletes selected for major international competitions, such as the Olympic Games, World Championships, and the Mediterranean Games. The research protocols for this study were examined and approved by the Review Board of the Institute of Medicine and Sports Science. The study design of the investigation was approved by the Review Board of the Institute of Sports Medicine and Science, the Italian National Olympic Committee, and by the ethic committee of Sapienza University of Roma, date of approval: 25 September 2024, code 0851/2024. All athletes included in this study were fully informed of the types and nature of the evaluation and signed the consent form, pursuant to Italian law and ISMS policy. All clinical data assembled from the study population are maintained in an institutional database. The research complies with the World Medical Association’s Code of Ethics (2024 revision of Declaration of Helsinki).

### 2.2. Study Population and Clinical Evaluation

This was a retrospective observational study performed in a single center. For this research, we enrolled elite athletes who were evaluated before the Olympic Games (both in summer and in winter) starting in London 2012 until the latest in Paris 2024. Specifically, athletes were engaged in the following 32 sporting disciplines, divided according to 2020 ESC guidelines on Sports Cardiology [9]: skill—including boules, skeet shooting, equestrian, golf, and sailing; power—including weightlifting, diving, artistic gymnastics, athletics (<800 mt), alpine skiing, snowboard, judo, Greek–Roman wrestling, and short distance swimming (<800 mt); mixed—including rhythmic gymnastics, fencing, boxing, volleyball, handball, tennis, soccer, badminton, basketball, beach volley, and water polo; and endurance—including rowing, long distance running (≥800 mt), long distance swimming (≥ 800 mt), biathlon, cross country skiing, and triathlon cycling.

Athletes underwent a thorough, multidisciplinary pre-participation evaluation, which comprised a complete physical exam, full blood tests analysis, resting electrocardiography (ECG), transthoracic echocardiography (TTE), and a cardiopulmonary exercise test (CPET). Exclusion criteria included athletes with clinically significant cardiovascular disease (including cardiomyopathies), those undergoing chronic medication therapy and individuals with incomplete medical records.

### 2.3. Anthropometric Measurements

Anthropometric measurements were obtained and recorded for each subject, and body mass index (BMI) was computed as weight (in kilograms) divided by height (in meters) squared. BSA was calculated using the Mosteller formula [13]. A standard 12-lead ECG was conducted with the subject in a supine position, and interpretation was performed in accordance with international criteria for ECG interpretation in athletes [14,15]. Blood pressure (BP) was assessed via non-invasive brachial cuff measurement while in a supine position at rest, according to 2024 ESC guidelines [16].

Cardiovascular (CV) risk factors were defined as follows: dyslipidemia was defined as LDL > 115 mg/dL [17]; cigarette smoking as regularly smokers of at least one cigarette per day [17]; office hypertension as systolic blood pressure (SBP) ≥ 140 mmHg and/or diastolic (DBP) ≥ 90 mmHg [16]; office elevated BP as SBP between 120 and 139 mmHg, or DBP between 70 and 89 mmHg [16]; diabetes as fasting glucose ≥ 126 mg/ dL, or current treatment with insulin or oral antidiabetic drugs [18]; hyperglycemia as glycemia values between 110 and 125 mg/dL [18]; and obesity was defined as a BMI ≥ 30 kg/m^2^ [17].

In order to compare athletes with extreme anthropometry and different phenotypes in relation to their functional and cardiac parameters, we arbitrarily grouped athletes according to BSA, as follows:

Group A: athletes with BSAs below the 5th percentile (a BSA < 1.62 m^2^, n = 25 for males and BSA < 1.41 m^2^, n = 19 for females);

Group B: athletes with BSAs between 25th and 75th percentiles (a BSA between 2.01 and 2.16, n = 368 for males, and a BSA between 1.71 and 1.84, n = 322 for females);

Group C: athletes with BSAs over the 95th percentile (a BSA > 2.42 m^2^, n = 41 for males, and a BSA > 2.09 m^2^, n = 26 for females).

### 2.4. Transthoracic Echocardiogram

The echocardiographic evaluation was conducted on participants while they were at rest, positioned in the left lateral decubitus position. Ultrasound data acquisition was performed utilizing a GE Vivid E9 ultrasound system equipped with a 4Vc phased array probe (GE Healthcare Vingmed Ultrasound AS, Strandpromenaden 45, 3183 Horten, Norway). A comprehensive 2D echocardiographic study was carried out, wherein cardiac images were captured in various cross-sectional planes employing established transducer positions. Conventional echocardiography was conducted according to American Society of Echocardiography (ASE) guidelines and the European Association of Cardiovascular Imaging (EACVI) [19,20,21]. The LV wall thickness was measured in the parasternal long-axis view. Left ventricular ejection fraction (LVEF) and left atrial (LA) volume index were determined using a modified Simpson’s method. The peak early diastolic velocity of LV inflow (E velocity), the late atrial diastolic velocity of LV inflow (A velocity), and the peak early diastolic velocity at the septal corner of the mitral annulus (e′) were recorded from the apical four-chamber view. Different types of LV remodeling were defined based on the measurements obtained, including normal geometry (NG) defined as LVM ≤ 95 g/m^2^ and RWT ≤ 0.42; concentric remodeling (CR) as LVM ≤ 95 g/m^2^ and RWT > 0.42; concentric hypertrophy (CH) as LVM > 95 g/m^2^ and RWT > 0.42; and eccentric hypertrophy (EH) as LVM > 95 g/m^2^ and RWT ≤ 0.42 [19,20,21].

### 2.5. Cardio-Pulmonary Exercise Test

We performed CPET using a cycle ergometer (COSMED, Rome, Italy). The protocol began with a one-minute rest period, followed by a two-minute warm-up with no resistance. Afterward, the workload increased progressively in increments of 15, 20, 25, or 30 watts, using a ramp protocol, tailored according to the athlete’s gender and sports discipline, continuing until exhaustion. All athletes included in the analysis met the RER criterion of >1.05 before their maximal VO_2_ measurements were recorded [22]. Continuous ECG monitoring and recording (Quark T12x, COSMED) took place during the warm-up, exercise, and subsequent recovery period, which lasted for five minutes. Additionally, we utilized a breath-by-breath metabolimeter (Quark CPET; COSMED) to measure oxygen consumption and carbon dioxide production throughout the entire cardiopulmonary assessment. The ventilatory threshold (VT) was identified using both the V-slope and ventilatory equivalents methods. The respiratory compensation point (RCP) was determined by analyzing the relationship between V’E and V’CO_2_ over time, following established standard criteria [22]. We recorded the following parameters at peak: VO_2_ in absolute (mL/min) and relative (mL/min/kg) values, power (expressed in watts), heart rate (HR), beats per minute, respiratory quotient (RQ), and oxygen pulse (VO_2_/HR). When reaching both the ventilatory threshold (V1/L1) and respiratory compensation threshold (V2/L2), measurements were conducted for: power (expressed in watts), VO_2_ (mL/min), and the slope of work efficiency (VO_2_/watts).

### 2.6. Statistical Analysis

Categorical variables were presented as frequencies and percentages, and their comparisons were conducted using Fisher’s exact test or the chi-squared test, as appropriate. For continuous variables, normality criteria were checked, and results were expressed as mean ± standard deviation (SD). Normal distribution of data was tested by the QQ graph method. Comparison between groups was performed using Student’s *t*-test or Mann–Whitney test, depending on the data distribution. A significance level of *p* < 0.05 was established. Dunn’s test and the pairwise comparison method were used to focus on differences between three groups. The pooled *p*-value for the comparison test is reported in the tables. If the pooled *p*-value was less than 0.05, a pairwise test was performed. Pairwise comparisons were considered significant if the *p*-value was less than 0.05. Statistical analysis was performed with STATA Statistics software for Windows (SE, version 17).

## 3. Results

We enrolled 2518 Olympic athletes (1384 males, 55%) of mean age 25.7 ± 5.2 years old, mean height 177 ± 11 cm, and mean BMI 23.2 ± 3.1 kg/m^2^.

The overall population of male athletes had a mean BSA of 2.02 ± 0.20. As previously described, three groups were created in both males and females according to BSA percentiles; namely, Group A included athletes below the 5th percentile (n = 25, BSA < 1.62), Group B athletes between the 25th and 75th percentiles (n = 368, BSA between 2.01 and 2.16), and Group C included athletes over 95th percentile (n = 41, BSA > 2.42).

**Figure 1:**
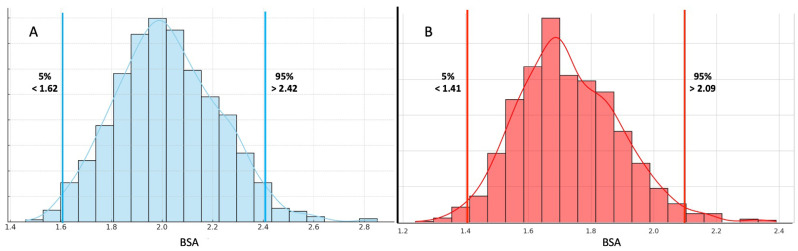
Normal distribution of BSA values in male (Panel (**A**)) and female (Panel (**B**)) athletes.

All female athletes had a mean BSA of 1.73 ± 0.16; Group A was composed of those below the 5th percentile (n = 19, BSA < 1.41), Group B athletes were between the 25th and 75th percentiles (n = 322, BSA between 1.71 and 1.84), and Group C included those over the 95th percentile (n = 26, BSA > 2.09), as shown in Figure 1.

Comparison of main clinical, anthropometric, and blood tests results are listed in Table 1.

Males with extreme anthropometry were clustered in specific sporting disciplines; we found a higher prevalence of mixed disciplines in Group C (63.4%, compared to 16% in Group A, and 34.5% in Group B, *p* = 0.0001), and a higher percentage of endurance was found in Group A (36% vs. 15.8% in Group B, and 7.3% in Group C, *p* = 0.003).

Specifically, athletes with a BSA over the 95th percentile were more commonly involved in basketball (n = 14), shot put (n = 8), volleyball (n = 6), judo (n = 3), handball (n = 3), water polo (n = 3), rowing (n = 2), and boxing (n = 2). On the other hand, those with a BSA under the 5th percentile practiced athletics (n = 10), boxing (n = 3), climbing (n = 2), artistic gymnastic (n = 2), golf (n = 2), taekwondo (n = 2), Greek–Roman wrestling (n = 2), weightlifting (n = 1), and equestrian (n = 1).

As expected, 31.7% of athletes in Group C presented a BMI > 30 kg/m^2^. From a clinical point of view, athletes with a higher anthropometry presented a higher cardiovascular risk profile characterized by higher rest SBP and DBP (both *p* < 0.0001), and peak BP values in exercise stress tests (SBP, *p* < 0.0001 and DBP, *p* = 0.0006), with a higher prevalence of office high blood pressure (HBP, 9.7% in Group C, *p* = 0.003). Moreover, a worse lipid profile was observed with higher serum TC levels in Group C (*p* = 0.037), higher LDL values (*p* = 0.005), higher LDL/HDL values (*p* = 0.001), and a higher prevalence of dyslipidemia (*p* = 0.031).

Then, functional and echocardiographic differences were assessed (Table 2). Globally, male athletes with extremely high anthropometry showed lower functional parameters in CPET compared to remaining groups (2.73 ± 0.6 W/kg in Group C vs. 3.5 ± 0.7 W/kg in Group B, and 3.87 ± 0.8 W/kg in Group C, *p* < 0.0001); data were confirmed also in VO_2_ max (35.2 ± 13.2 mL/min/Kg in C, 44 ± 7.1 mL/min/Kg in B, and 47.8 ± 7.3 mL/min/kg in A, *p* < 0.0001).

In TTE, athletes with a BSA over the 95th percentile presented with a larger LV, with mean 59 ± 3 mm vs. 55 ± 2.9 mm in Group B, and 51.1 ± 3.1 mm in Group A, *p* < 0.0001 and LVEDV (*p* < 0.0001). Athletes with high extreme anthropometry also showed a larger IVS and PWT (both *p* < 0.0001), and a higher LVM (*p* < 0.0001). Finally, no significant differences in LV geometry were observed among athletes with different anthropometry, with similar rates of EH prevalence (24% in Group A, 17.7% in Group B, and 14.6% in Group C, *p* = 0.501).

Regarding female athletes, also in this category, a higher prevalence of mixed disciplines was found in athletes with a higher body size (73.1% in Group C vs. 34.8% in Group B, and 10.5% in Group A, *p* < 0.0001) and a higher percentage of endurance athletes was found in those with lowest anthropometry (26.3% in Group A vs. 16.1% in Group B, and no cases in Group C, *p* = 0.037).

Athletes with the lowest BSAs practiced most commonly the following sporting disciplines: rhythmic gymnastics (n = 7), athletics (n = 5), artistic gymnastic (n = 3), equestrian (n = 1), archery (n = 1), weightlifting (n = 1), and climbing (n = 1). On the other side, females with the highest BSAs were involved in volleyball (n = 9), water polo (n = 5), shot put (n = 4), basketball (n = 3), judo (n = 3), boules (n = 1), and target shooting (n = 1).

As well as in males, also in females were significant anthropometric differences registered, in height, weight, and BMI (all *p* < 0.0001), with a 42.3% of athletes in Group C presenting a BMI > 30 kg/m^2^. Clinically, females with the largest BSAs presented higher BP values both at rest and during the exercise stress test (*p* < 0.0001 for SBP and DBP at rest, and *p* < 0.0001 for SBP at peak and *p* = 0.0005 for DPB at peak of exercise stress test), with higher prevalence (but not a statistically significant difference) of office HBP, 3.8%, *p* = 0.196. Differing from male athletes, no significant differences were found in lipid profiles (TC, *p* = 0.468; LDL, *p* = 0.384; LDL/HDL ratio, *p* = 0.057) with similar rates of dyslipidemia (*p* = 0.609).

In functional evaluation, also in female athletes was a trend in functional parameters recorded (2.34 ± 0.5 W/kg in Group C vs. 3.17 ± 0.6 W/kg in Group B, and 3.42 ± 0.8 W/kg in Group A, *p* < 0.0001). Also, VO_2_ max was lower in those with a BSA over the 95th percentile (31.5 ± 7.4 mL/min/kg vs. 39.9 ± 6.7 mL/min/kg in Group B, and 44.7 ± 5 mL/min/kg in Group A, *p* = 0.0001). In TTE, athletes with a BSA over the 95th percentile presented a larger LV, with 53.1 ± 2.8 vs. 50.2 ± 3 mm in Group B, and 46.5 ± 2.4 mm in Group A, *p* < 0.0001 and LVEDV (*p* < 0.0001). Athletes with high extreme anthropometry also showed a thicker IVS and PWT (both *p* < 0.0001), and a higher LVM (*p* < 0.0001). Finally, no significant differences in LV geometry were observed among athletes with different anthropometries, with similar rates of EH prevalence (21% in Group A, 28% in Group B, and 15.4% in Group C, *p* = 0.501).

## 4. Discussion

Our study highlights the relevance of anthropometric characteristics, as simply expressed by BSA, in shaping the morpho-functional and cardiometabolic profile of elite athletes. While sport classification systems remain clinically helpful, they may insufficiently capture the wide inter-individual variability in cardiac remodeling, functional performance, and cardiometabolic risk, which exist within and across sport disciplines. Our data suggests that shifting the analytical focus from sport discipline alone to valorize also body size offers valuable additional physiological and clinical insights.

In particular, the rationale for adopting an anthropometric-based approach lies in the established influence of BSA and BMI on cardiac morphology and function. In fact, previous studies have shown that extent of cardiac remodeling in athletes can be explained largely by body size [23,24]. Specifically, Pelliccia et al. [24] showed, in a cohort of 1111 Olympic athletes, that the combination of age, sex and BMI explain more than 60% of the LV mass, while sport alone averages at 14%.

In our cohort, we found that athletes at the extremes of BSA distribution, particularly those above the 95th percentile (Group C), demonstrated significantly different cardiovascular phenotypes compared to their peers, even when practicing similar sports.

Furthermore, our findings revealed a non-random distribution of sport disciplines across BSA categories; while athletes with a low BSA were predominantly involved in endurance disciplines, those with a high BSA were more frequently engaged in mixed and power sports [25]. This suggests that certain sports inherently select distinct anthropometric traits, reinforcing the physiological divergence that sport-based classification alone may obscure, as previously suggested by Riding et al. [26], who demonstrated that extreme anthropometric traits, common in certain disciplines, significantly affect cardiac morphology and risk profile independently of training or sport practice.

Moreover, athletes with a higher BSA exhibited a more adverse cardiometabolic and hemodynamic profile, including higher resting and peak blood pressure, increased prevalence of hypertension, and unfavorable lipid parameters (especially in males). Interestingly, the unfavorable lipid profile observed in male athletes with higher BSAs was not mirrored in their female counterparts. This difference may be partially explained by the protective role of estrogens, which are known to positively influence lipid metabolism. Estrogenic effects may attenuate the emergence of metabolic alterations associated with larger body size, thereby blunting sex-related differences in lipid profiles among female athletes [27].

Another interesting point of our study is that athletes with higher BSAs demonstrated functionally lower relative aerobic capacity (W/kg and VO_2_ max/kg/min), despite having larger absolute cardiac dimensions. These features may reflect an increased hemodynamic load, suboptimal work efficiency, or intrinsic limitations in cardiorespiratory performance among individuals with larger body size.

This is in line with the current literature. Notably, Maciejczyk et al. demonstrated that high body mass, regardless of the cause, decreases relative VO_2_ max [28].

From a morphological point of view, although LV geometry patterns did not significantly differ among BSA groups, athletes with a high BSA showed a substantially greater wall thickness and LV mass, which may reflect the physiological adaptation to a higher BSA, but could, in some cases, mimic pathological remodeling.

Taken together, these findings underscore the need to consider anthropometric characteristics as fundamental components in the clinical assessment of athletes, in particular in assessing the athlete’s heart and CV risk profile. Recognizing extreme body size as a potential modifier of both performance metrics and cardiovascular risk may improve the precision of sports’ cardiology evaluations. BSA should not merely be a variable for indexation but rather a lens through which morpho-functional and metabolic data are interpreted.

This study also raises questions about the adequacy of current athlete classification systems and whether complementary stratification methods, such as BSA-based grouping, should be integrated into routine clinical and research practices.

### Limitations

This study has several limitations. Firstly, its retrospective and observational design inherently limits its ability to establish causal relationships between BSA and the observed morpho-functional and cardiometabolic profiles. Secondly, although the cohort included a large and heterogeneous population of Olympic athletes across a wide range of sports, the sample sizes in extreme BSA categories were relatively small, potentially affecting statistical power and generalizability. Third, the use of BSA percentiles for group stratification, while useful to highlight extreme phenotypes, was arbitrarily defined and not based on established reference values for athletes. Moreover, all CPETs were performed on a cycle ergometer to ensure methodological consistency across a wide variety of sports disciplines. However, this choice may not fully reflect the “true” VO_2_ max values for athletes whose primary activity involves running or other weight-bearing endurance sports. However, it is important to emphasize that the aim of our study was not to provide reference VO_2_ max values for elite athletes, but rather to offer an indicative measure of cardiorespiratory fitness in the context of variability related to BSA. Lastly, our findings may not be applicable to non-elite athletic populations, recreational athletes, or individuals with different ethnic or training backgrounds.

## 5. Conclusions

In this large cohort of Olympic athletes, BSA emerged as a relevant determinant of cardiovascular, functional, and metabolic profiles. Athletes with higher BSAs demonstrated lower relative aerobic capacity and a more adverse cardiometabolic profile, especially among males, despite displaying larger cardiac dimensions. Our findings emphasize the importance of considering anthropometric traits, particularly at the extremes of body size, as independent modulators of athletes’ physiology. These data support the integration of anthropometric assessment, beyond simple indexation, as a primary step in the clinical evaluation of elite athletes.

## Figures and Tables

**Table 1:** Main clinical and anthropometric parameters differences among the groups in both genders.

MALES, n = 434	Group A	Group B	Group C	P Pooled	P Pairwise
Percentile	5%	25–75%	95%		
n	25	368	41		
Age, years	26.3 ± 5.3	26.3 ± 5.2	27.4 ± 5.4	0.410	-
Skill, n (%)	3 (12)	44 (12)	0 (0)	0.064	-
Power, n (%)	9 (36)	139 (37.8)	13 (31.7)	0.744	-
Mixed, n (%)	4 (16)	127 (34.5)	26 (63.4)	0.0001	**A vs. C, *****p***** < 0.0001; B vs. C, *****p***** = 0.0003;** A vs. B, *p* = 0.057.
Endurance, n (%)	9 (36)	58 (15.8)	2 (4.9)	0.003	**A vs. B, *****p***** = 0.009; A vs. C, *****p***** = 0.0007;** B vs. C, *p* = 0.062.
Afro-Caribbean, n (%)	5 (20)	19 (5.2)	3 (7.3)	0.011	**A vs. B, *****p***** = 0.002;** A vs. C, *p* = 0.129; B vs. C, *p* = 0.563.
Height, cm	167.2 ± 7	184.9 ± 5.2	200.2 ± 7.7	<0.0001	**All, ** ***p*** ** < 0.0001.**
Weight, kg	55.2 ± 4.7	84.1 ± 4.8	114.8 ± 12.9	<0.0001	**All, ** ***p*** ** < 0.0001.**
BSA	1.58 ± 0.03	2.10 ± 0.05	2.52 ± 0.10	<0.0001	**All, ** ***p*** ** < 0.0001.**
BMI, kg/m^2^	19.8 ± 2.3	24.6 ± 2.2	28.9 ± 5.1	<0.0001	**All, ** ***p*** ** < 0.0001.**
BMI > 30 kg/m^2^	0 (0)	5 (1.3)	13 (31.7)	<0.0001	**A vs. C, *****p***** = 0.001; B vs. C, *****p***** < 0.0001;** A vs. B, *p* = 0.557.
Rest SBP, mmHg	110.4 ± 13	117.4 ± 9.6	123.8 ± 10.4	<0.0001	**A vs. B, ** ***p*** ** = 0.001; A vs. C, ** ***p*** ** = 0.0001; B vs. C, ** ***p*** ** = 0.0004.**
Rest DBP, mmHg	68.2 ± 7.5	72.2 ± 7.2	77.3 ± 7.5	<0.0001	**A vs. B, ** ***p*** ** = 0.014; A vs. C, ** ***p*** ** < 0.0001; B vs. C, ** ***p*** ** = 0.0002.**
Office Hypertension, n (%)	0 (0)	6 (1.6)	4 (9.7)	0.003	**B vs. C, *****p***** = 0.001;** A vs. B, *p* = 0.520; A vs. C, *p* = 0.107.
Rest HR, bpm	58.4 ± 11.4	63.6 ± 12.6	67.1 ± 18.8	0.062	-
Glycemia, mg/dL	92 ± 6.5	92 ± 7.1	92.3 ± 12.3	0.971	-
GI, n (%)	3 (12)	42 (11.4)	5 (12.2)	0.988	-
TC, mg/dL	165.7 ± 25	173 ± 32.1	184.5 ± 29.6	0.037	**A vs. C, *****p***** = 0.011; B vs. C, *****p***** = 0.028;** A vs. B, *p* = 0.270.
HDL, mg/dL	65.8 ± 19	62.1 ± 15.3	59.6 ± 17.4	0.296	-
LDL, mg/dL	85.9 ± 16.7	96.6 ± 28	107.9 ± 25.4	0.005	**A vs. C, *****p***** = 0.0003; B vs. C, *****p***** = 0.014;** A vs. B, *p* = 0.059.
LDL/HDL	1.38 ± 0.4	1.7 ± 0.7	1.96 ± 0.7	0.001	**A vs. B, ** ***p*** ** = 0.045; A vs. C, ** ***p*** ** = 0.0004; B vs. C, ** ***p*** ** = 0.004.**
Dyslipidemia, n (%)	1 (4)	41 (11.1)	13 (31.7)	0.031	**A vs. B, *****p***** = 0.028; A vs. C, *****p***** = 0.007;** B vs. C, *p* = 0.194.
TG, mg/dL	74.4 ± 44.7	75.7 ± 39.7	88.7 ± 58	0.164	-
**FEMALES, n= 367**	**Group A**	**Group B**	**Group C**	**P Pooled**	**P Pairwise**
**Percentile**	**5%**	**25–75%**	**95%**		
**n**	**19**	**322**	**26**		
Age, years	25.2 ± 7.1	24.8 ± 4.4	25.8 ± 5.4	0.570	-
Skill, n (%)	2 (10.5)	42 (13)	2 (7.7)	0.705	-
Power, n (%)	10 (52.6)	116 (36)	5 (19.2)	0.065	-
Mixed, n (%)	2 (10.5)	112 (34.8)	19 (73.1)	<0.0001	**A vs. B, ** ***p*** ** = 0.029; A vs. C, ** ***p*** ** < 0.0001; B vs. C, ** ***p*** ** < 0.0001.**
Endurance, n (%)	5 (26.3)	52 (16.1)	0 (0)	0.037	**A vs. C, *****p***** = 0.004; B vs. C, *****p***** = 0.026;** A vs. B, *p* = 0.249.
Afro-Caribbean, n (%)	0 (0)	7 (2.2)	3 (11.5)	0.013	**B vs. C, *****p***** = 0.005;** A vs. B, *p* = 0.517; A vs. C, *p* = 0.131.
Height, cm	152.3 ± 5.7	171.6 ± 4.9	183.2 ± 8.2	<0.0001	**All, ** ***p*** ** < 0.0001.**
Weight, kg	44.7 ± 3.1	66.3 ± 4.3	94.8 ± 11.6	<0.0001	**All, ** ***p*** ** < 0.0001.**
BSA	1.37 ± 0.04	1.77 ± 0.04	2.18 ± 0.08	<0.0001	**All, ** ***p*** ** < 0.0001.**
BMI, kg/m^2^	19.3 ± 2.1	22.6 ± 1.9	28.6 ± 5.3	<0.0001	**All, ** ***p*** ** < 0.0001.**
BMI > 30 kg/m^2^	0 (0)	1 (0.3)	11 (42.3)	<0.0001	**A vs. C, *****p***** = 0.001; B vs. C, *****p***** < 0.0001;** A vs. B, *p* = 0.807.
Rest SBP, mmHg	95 ± 9.7	108.3 ± 9.9	115.4 ± 9.7	<0.0001	**A vs. B, *****p***** < 0.0001; A vs. C, *****p***** < 0.0001; B vs. C, *****p***** = 0.0007 **.
Rest DBP, mmHg	61.1 ± 6.6	67.1 ± 7.6	71.8 ± 8.2	<0.0001	**A vs. B, ** ***p*** ** = 0.001; A vs. C, ** ***p*** ** < 0.0001; B vs. C, ** ***p*** ** = 0.003.**
Office Hypertension, n (%)	0 (0)	2 (0.6)	1 (3.8)	0.196	-
Rest HR, bpm	69.7 ± 16.6	63.9 ± 12.6	65.6 ± 14.8	0.186	-
Glycemia, mg/dL	89.6 ± 5	89.8 ± 6.7	89.6 ±8.4	0.980	-
GI, n (%)	0 (0)	18 (5.6)	2 (7.7)	0.508	-
TC, mg/dL	183.2 ± 29	176.8 ± 30.1	182.1 ± 21.2	0.468	-
HDL, mg/dL	72.8 ± 20.1	69.5 ± 14.7	66.7 ± 17.6	0.420	-
LDL, mg/dL	96 ± 25.9	94.1 ± 24.2	100.8 ± 19.2	0.384	-
LDL/HDL	1.45 ± 0.6	1.42 ± 0.5	1.67 ± 0.7	0.057	-
Dyslipidemia, n (%)	4 (21)	60 (18.6)	7 (26.9)	0.609	-
TG, mg/dL	80.5 ± 24.3	69.4 ± 31.1	76.3 ± 27.5	0.186	-

**Abbreviations**: BMI: body mass index; BSA: body surface area; DBP: diastolic blood pressure; GI: glucose intolerance; HDL: high density lipoprotein; HR: heart rate; LDL: low density lipoprotein; SBP: systolic blood pressure; TC: total cholesterol; and TG: triglycerides.

**Table 2:** Comparison of main functional parameters in CPET and echocardiographic measurements among the three groups.

MALES, n = 434	Group A	Group B	Group C	P Pooled	P Pairwise
Percentile	5%	25–75%	95%		
n	25	368	41		
W/kg	3.87 ± 0.8	3.5 ± 0.7	2.73 ± 0.6	<0.0001	**A vs. B, ** ***p*** ** = 0.005; A vs. C, ** ***p*** ** < 0.0001; B vs. C, ** ***p*** ** < 0.0001.**
Peak SBP, mmHg	166.4 ± 19.7	186.7 ± 18.9	191.5 ± 18.8	<0.0001	**A vs. B, *****p***** < 0.0001; A vs. C, *****p***** < 0.0001**; B vs. C, *p* = 0.124.
Peak DBP, mmHg	75.4 ± 7.1	77.9 ± 8.6	82.9 ± 8.4	0.0006	**A vs. C, *****p***** = 0.0006; B vs. C, *****p***** = 0.0006;** A vs. B, *p* = 0.156.
VO_2_ max, mL/min	2657.3 ± 484.6	3676.7 ± 603.6	3926.71 ± 580.7	<0.0001	**A vs. B, *****p***** < 0.0001; A vs. C, *****p***** < 0.0001;** B vs. C, *p* = 0.1099.
VO_2_ max, mL/min/kg	47.8 ± 7.3	44 ± 7.1	35.2 ± 13.2	<0.0001	**A vs. C, *****p***** < 0.0001; B vs. C, *****p***** < 0.0001;** A vs. B, *p* = 0.088.
VO_2_ @ VT1, mL/min	1467.2 ± 448.4	2281.7 ± 719.2	2396.1 ± 800.7	0.001	**A vs. B, *****p*****= 0.0003; A vs. C, *****p***** = 0.002;** B vs. C, *p* = 0.544
VO_2_ @ VT2, mL/min	2282.3 ± 423.6	3165.5 ± 795	3343.1 ± 526.4	0.001	**A vs. B, *****p*****= 0.0008; A vs. C, *****p***** < 0.0001;** B vs. C, *p* = 0.44
O_2_ pulse, mL/beat	18 ± 5.5	22.4 ± 4	24.7 ± 4.6	0.0004	**A vs. B, ** ***p*** ** = 0.001; A vs. C, ** ***p*** ** = 0.002; B vs. C, ** ***p*** ** = 0.032.**
LVEDD, mm	51.1 ± 3.1	55 ± 2.9	59 ± 3	<0.0001	**All, ** ***p*** ** < 0.0001.**
IVS, mm	9.04 ± 0.8	10 ± 0.9	10.4 ± 0.9	<0.0001	**A vs. B, ** ***p*** ** < 0.0001; A vs. C, ** ***p*** ** < 0.0001; B vs. C, ** ***p*** ** = 0.019.**
PWT, mm	8.56 ± 0.8	9.62 ± 1	10.1 ± 1.2	<0.0001	**A vs. B, ** ***p*** ** < 0.0001; A vs. C, ** ***p*** ** < 0.0001; B vs. C, ** ***p*** ** = 0.003.**
LVEDV, mL	114.4 ± 24.3	154.2 ± 32	192.2 ± 39.2	<0.0001	**All, ** ***p*** ** < 0.0001.**
EF, %	63.7 ± 5.8	63.5 ± 5.5	62.7 ± 5	0.667	-
RWT	0.33 ± 0.02	0.35 ± 0.04	0.34 ± 0.04	0.084	-
LVM, g	160.5 ± 29.2	209.1 ± 36.5	249.3 ± 41.9	<0.0001	**All, ** ***p*** ** < 0.0001.**
LVEDDi, mm/BSA	32.3 ± 2.2	26.5 ± 1.4	23.5 ± 1.6	<0.0001	**All, ** ***p*** ** < 0.0001.**
LVEDVi, mL/BSA	72.3 ± 15.8	74.3 ± 15.3	76.4 ± 15.6	0.565	-
LVMi, g/BSA	101.5 ± 19.4	100.6 ± 17.3	99 ± 16.2	0.807	-
EH, n (%)	6 (24)	65 (17.7)	6 (14.6)	0.501	-
CR, n (%)	0 (0)	4 (1.1)	0 (0)	0.677	-
CH, n (%)	0 (0)	2 (0.5)	0 (0)	0.822	-
NG, n (%)	19 (76)	297 (80.7)	35 (85.4)	0.475	-
LAVi, mL/BSA	17.7 ± 6.6	22.3 ± 6.9	26 ± 7.4	0.0001	**A vs. B, ** ***p*** ** = 0.004; A vs. C, ** ***p*** ** = 0.0002; B vs. C, ** ***p*** ** = 0.003.**
RVEDDi, mm/BSA	23.2 ± 2.3	19.5 ± 2.3	17.2 ± 1.3	<0.0001	**All, ** ***p*** ** < 0.0001.**
E/A	1.56 ± 0.8	1.45 ± 0.7	1.28 ± 0.6	0.204	-
**FEMALES, n = 367**	**Group A**	**Group B**	**Group C**	**P Pooled**	**P Pairwise**
**Percentile**	**5%**	**25–75%**	**95%**		
**n**	**19**	**322**	**26**		
W/kg	3.42 ± 0.8	3.17 ± 0.6	2.34 ± 0.5	<0.0001	**A vs. C, *****p***** < 0.0001; B vs. C, *****p***** < 0.0001**; A vs. B, *p* = 0.105.
Peak SBP, mmHg	147.5 ± 15.8	166.6 ± 15.4	176 ± 17	<0.0001	**A vs. B, ** ***p*** ** < 0.0001; A vs. C, ** ***p*** ** < 0.0001; B vs. C, ** ***p*** ** = 0.003.**
Peak DBP, mmHg	70.3 ± 10.1	74.9 ± 10	79.8 ± 7.9	0.0005	**A vs. C, *****p***** = 0.001; B vs. C, *****p***** = 0.015**; A vs. B, *p* = 0.051.
VO_2_ max, mL/min	1994.8 ± 333.2	2645.4 ± 419.3	2830.3 ± 557.1	0.001	**A vs. B, *****p***** = 0.0008; A vs. C, *****p***** = 0.011;** B vs. C, *p* = 0.172.
VO_2_ max, mL/min/kg	44.7 ± 5	39.9 ± 6.7	31.5 ± 7.4	0.0001	**A vs. C, *****p***** = 0.004; B vs. C, *****p***** = 0.0001;** A vs. B, *p* = 0.109.
VO_2_ @ VT1, mL/min	1217.6 ± 230.6	1695.9 ± 478.1	1769.9 ± 456.2	0.075	**-**
VO_2_ @ VT2, mL/min	1595.5 ± 268.2	2241.5 ± 653.3	2491.7 ± 560.4	0.067	**-**
O_2_ pulse, mL/beat	11.5 ± 1.5	15.5 ± 2.6	17.5 ± 3.1	0.0002	**A vs. B, ** ***p*** ** = 0.0007; A vs. C, ** ***p*** ** = 0.001; B vs. C, ** ***p*** ** = 0.017.**
LVEDD, mm	46.5 ± 2.4	50.2 ± 3	53.1 ± 2.8	<0.0001	**All, ** ***p*** ** < 0.0001.**
IVS, mm	7.84 ± 0.9	8.9 ± 0.9	9.27 ± 0.9	<0.0001	**A vs. B, ** ***p*** ** < 0.0001; A vs. C, ** ***p*** ** < 0.0001; B vs. C, ** ***p*** ** = 0.045.**
PWT, mm	7.4 ± 0.9	8.41 ± 1	8.81 ± 1	<0.0001	**A vs. B, *****p***** < 0.0001; A vs. C, *****p***** < 0.0001;** B vs. C, *p* = 0.051.
LVEDV, mL	86.5 ± 18	110.1 ± 20.7	139.4 ± 19.9	<0.0001	**All, ** ***p*** ** < 0.0001.**
EF, %	64.4 ± 4.9	63.8 ± 4.8	64.1 ± 5.8	0.847	-
RWT	0.32 ± 0.03	0.33 ± 0.03	0.33 ± 0.04	0.090	-
LVM, g	113.5 ± 25.9	152.5 ± 30	176.4 ± 28.5	<0.0001	**A vs. B, ** ***p*** ** < 0.0001; A vs. C, ** ***p*** ** < 0.0001; B vs. C, ** ***p*** ** = 0.0001.**
LVEDDi, mm/BSA	34.1 ± 2	28.4 ± 1.8	24.4 ± 1.4	<0.0001	**All, ** ***p*** ** < 0.0001.**
LVEDVi, mL/BSA	63.3 ± 12.8	62.2 ± 11.6	64 ± 8.1	0.674	-
LVMi, g/BSA	83.2 ± 19.4	86.2 ± 16.8	80.8 ± 10.9	0.700	-
EH, n (%)	4 (21)	90 (28)	4 (15.4)	0.120	-
CR, n (%)	0 (0)	2 (0.6)	0 (0)	0.989	-
CH, n (%)	0 (0)	0 (0)	0 (0)	1.000	-
NG, n (%)	15 (78.9)	230 (71.4)	22 (84.6)	0.120	-
LAVi, mL/BSA	20.6 ± 5.6	20.5 ± 5.9	20.9 ± 6.5	0.966	-
RVEDDi, mm/BSA	23.2 ± 2.5	19.7 ± 2.4	17.8 ± 1.7	<0.0001	**A vs. B, ** ***p*** ** = 0.0002; A vs. C, ** ***p*** ** < 0.0001; B vs. C, ** ***p*** ** = 0.007.**
E/A	1.47 ± 0.5	1.46 ± 0.7	1.37 ± 0.6	0.792	-

**Abbreviations**: BSA: body surface area; CH: concentric hypertrophy; CR: concentric remodeling; DBP: diastolic blood pressure; EF: ejection fraction; EH: eccentric hypertrophy; IVS: interventricular septum; LAVi: left atrial volume indexed; LVEDD: left ventricle end-diastolic diameter; LVEDDi: left ventricle end-diastolic diameter indexed; LVEDV: left ventricle end-diastolic volume; LVEDVi: left ventricle end-diastolic volume indexed; LVM: left ventricular mass; LVMi: left ventricular mass indexed; NG: normal geometry; O_2_ pulse: oxygen pulse; PWT: posterior wall thickness; RVEDDi: right ventricle end-diastolic diameter indexed; RWT: relative wall thickness; SBP: systolic blood pressure; VO_2_ max: maximal oxygen consumption; VT1: first ventilatory threshold; VT2: second ventilatory threshold; and W: watts.

## Data Availability

The data presented in this study are available on request from the corresponding author. The data are not publicly available due to privacy or ethical restrictions.
